# Kann ein Sturz auf die Schulter (Direktanprall) einen Riss der Rotatorenmanschette verursachen?

**DOI:** 10.1007/s00132-024-04474-x

**Published:** 2024-02-19

**Authors:** Richard W. Nyffeler, Alois Lustenberger, Philipp Bissig

**Affiliations:** 1 Orthopädie Sonnenhof KLG, Salvisbergstrasse 4, 3006 Bern, Schweiz; 2 Campus Stiftung Lindenhof Bern (Campus SLB), Salvisbergstrasse 4, 3006 Bern, Schweiz; 3 Hirslanden Klinik Linde, Blumenrain 105, 2501 Biel, Schweiz; 4 Orthomed, Orthopädische Chirurgie Biel-Seeland, Bifangweg 1, 3270 Aarberg, Schweiz

**Keywords:** Massenruptur, Trauma, Unfallkausalität, Akut, Degenerativ, Massive tear, Trauma, Cause, Acute, Degenerative

## Abstract

Die Frage, ob ein Sturz direkt auf die Schulter eine Rotatorenmanschettenruptur verursachen kann, beschäftigt Ärzte (und Gerichte) seit vielen Jahren. Gutachter, welche sich auf die versicherungsmedizinische Literatur stützen, lehnen die Unfallkausalität in der Regel ab. Wissenschaftliche Untersuchungen dazu gibt es nicht. Der untenstehende Bericht beschreibt einen typischen Fall, bei dem ein Sturz direkt auf die Schulter eine massive Rotatorenmanschettenruptur verursacht hat.

## Abstract (en)

The question of whether a fall directly onto the shoulder can cause a rotator cuff tear has occupied doctors (and courts) for many years. Experts who rely on the medical insurance literature usually reject the causality of the incident. There are no scientific studies on this. The report below describes a typical case in which a fall directly onto the shoulder caused a massive rotator cuff tear.

## Einleitung

Rotatorenmanschettenrupturen gehören zu den häufigsten Pathologien der Schulter. Dass deren Prävalenz mit dem Alter zunimmt, ist unbestritten. Über die genauen Zahlen und über die Ursachen herrscht jedoch Uneinigkeit. Große epidemiologische Untersuchungen fehlen, und bei der Frage nach der Ätiologie gehen die Meinungen weit auseinander. In der angelsächsischen Literatur sind traumatische Läsionen gut vertreten und haben einen Anteil von bis zu 40 % [8]. In der deutschsprachigen versicherungsmedizinischen Literatur wird dagegen die Meinung vertreten, dass es eine isolierte unfallbedingte Zusammenhangstrennung der Rotatorenmanschette nicht gibt [6] oder dass eine solche selten ist [2]. Zudem wird von vielen Autoren proklamiert, dass nur wenige, genau definierte Verletzungsmechanismen geeignet sind, eine Sehnenläsion zu verursachen. Als ungeeignet gilt ein Sturz direkt auf die Schulter [1, 2, 5]. Mit diesem Beitrag wollen wir zeigen, dass diese Aussage nicht immer zutrifft. Der betroffene Patient hat eingewilligt, dass seine Krankengeschichte und seine MRT-Bilder für die Publikation in einer wissenschaftlichen Zeitschrift verwendet werden.

## Fallbeschreibung

**Figure 1:**
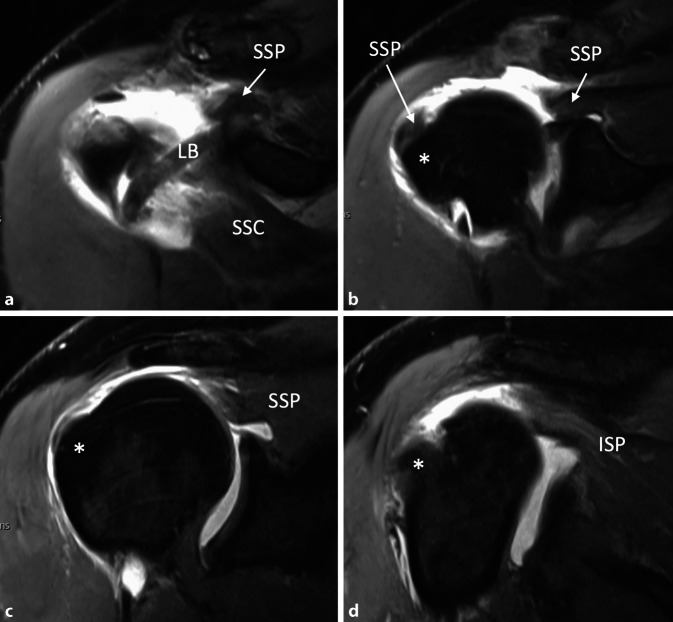

Ein 50-jähriger, gesunder, 1,80 m großer und 110 kg schwerer Gebäudetechniker rutschte beim Aussteigen aus seinem Lieferwagen auf dem Trittbrett aus und stürzte direkt auf seine rechte, dominante Schulter. Er verspürte sofort starke Schmerzen und konnte den Arm nicht mehr aktiv anheben. Er suchte deshalb am darauffolgenden Tag die Notfallaufnahme eines öffentlichen Spitals in Bern auf. Die Ärzte notierten in ihrem Bericht, dass der Patient direkt auf die Schulter gestürzt sei und sich zum Ausschluss einer Fraktur vorstellte. In der klinischen Untersuchung fanden sie weder eine Fehlstellung noch eine Schwellung und auch kein Hämatom. Die passive Elevation war ab 45° schmerzhaft. Auf eine Prüfung der Rotatorenmanschette verzichteten sie aufgrund der schmerzbedingt reduzierten Beurteilbarkeit. Eine Fraktur wurde mit konventionellen Röntgenaufnahmen in drei Ebenen ausgeschlossen. Der Arm wurde mit einer Schlinge ruhiggestellt, und zur Schmerzbehandlung verordneten die Ärzte Ibuprofen, Paracetamol und Metamizol. Bei der Verlaufskontrolle nach 3 Tagen waren die Schmerzen geringer, der Bewegungsumfang und die Kraft waren subjektiv aber immer noch deutlich vermindert. Es wurde deshalb eine Weiterabklärung mit einer Arthro-MRT-Untersuchung in die Wege geleitet. Diese fand 6 Tage nach dem Sturz statt und zeigte eine vollständige Ruptur der Subscapularis‑, Supraspinatus- und Infraspinatussehne sowie eine Luxation der langen Bizepssehne aus dem Sulcus intertubercularis heraus. Die Supraspinatussehne war bis zum Glenoid (Patte III), die Infraspinatussehne bis über den Zenit (Patte II) und die Subscapularissehne um etwa 1 cm (Patte I) retrahiert. Die Rotatorenmanschettenmuskulatur wies eine gute Trophik auf und enthielt keine fettige Infiltration (Abb. 1, 2 und 3). Hinweise für eine stattgehabte Schulterluxation (Hill-Sachs-Impressionsfraktur oder „reversed“ Hill-Sachs-Läsion) fehlten, ebenso wie ein Knochenödem. Am Tuberculum majus war noch ein kurzer Sehnenstummel sichtbar. Der Patient wurde uns zur operativen Behandlung zugewiesen. Er versicherte, bis zum erwähnten Ereignis nie Probleme mit seiner Schulter gehabt und auch keine anderen Stürze erlitten zu haben. Die Sehnen wurden 12 Tage nach dem Trauma offen reponiert und mit Fadenankern zweireihig fixiert. Der Sehnenstummel am Tuberculum majus war etwa 1 cm lang und wies Einblutungen auf. Er wurde belassen und mit der reponierten Rotatorenmanschette vernäht. Auf eine Biopsie wurde verzichtet. Anschließend wurde der Arm für 6 Wochen auf einem Abduktionskissen gelagert. In dieser Zeit waren nur passive Bewegungen erlaubt. Nach 6 Wochen begannen wir mit aktiven Bewegungen mit kurzem Hebelarm, nach 3 Monaten mit vorsichtigem Kraftaufbau. Eine Kontroll-MRT 5 Monate nach der Operation zeigte eine intakte Rotatorenmanschette, mit einer Ausdünnung der Supraspinatussehne und einer leichten fettigen Infiltration der zugehörigen Muskeln Grad I nach Goutallier (Abb. 4).

**Figure 2:**
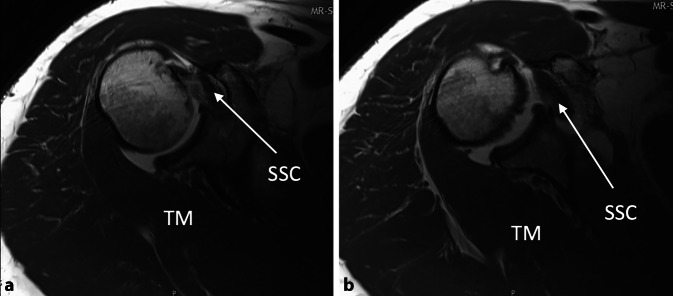

**Figure 3:**
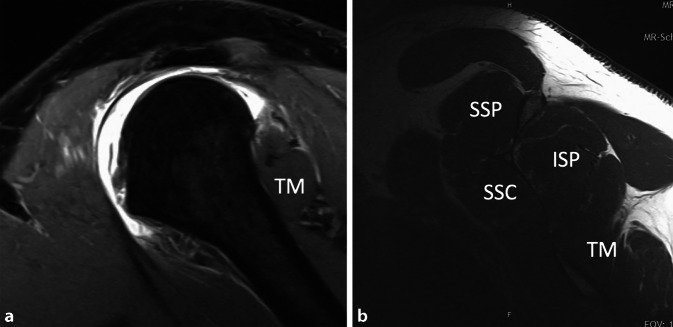

**Figure 4:**
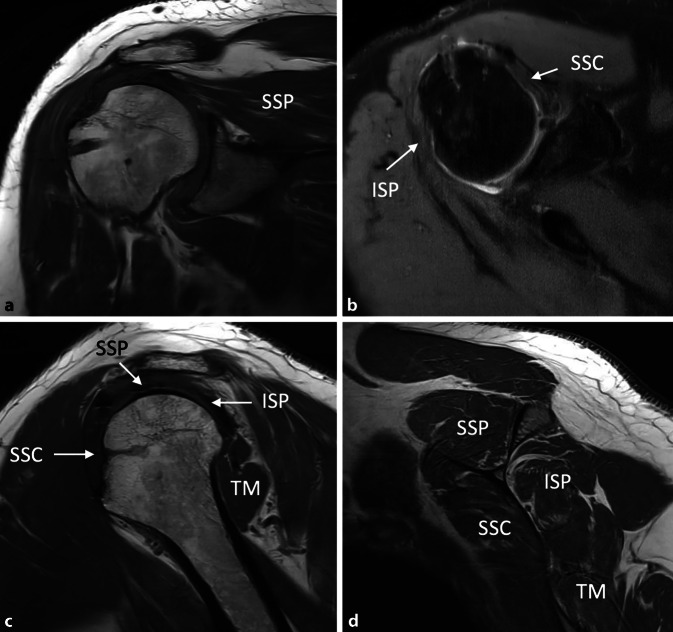

## Diskussion

In einer Zusammenstellung von 67 Peer-Reviewed-Studien mit insgesamt 4061 unfallbedingten Rotatorenmanschettenrupturen war ein Sturz die häufigste Ursache (18 %) für einen traumatischen Sehnenriss [9]. Als weitere Gründe wurden mit absteigender Häufigkeit folgende Ereignisse genannt: Schulterluxation (14 %), plötzlicher, kräftiger Zug am Arm (7 %), Sportverletzungen (4 %), Schlag gegen die Schulter oder Direktanprall (3 %), Verkehrsunfall (2 %), Hyperextension, forcierte Abduktion und Außenrotation oder das Festhalten an einem Geländer (2 %), und das Heben eines schweren Gegenstandes oder das Auffangen eines fallenden Objektes (1 %). In 49 % der Fälle wurde der Verletzungsmechanismus nicht näher bezeichnet. Eine biomechanische Begründung für die Sehnenrisse wurde nur in zwei der 67 untersuchten Arbeiten gegeben. Lindblom [4] und Matsen [7] postulierten, dass beim Sturz auf die ausgestreckte Hand oder den Ellbogen eine forcierte Adduktion im Glenohumeralgelenk auftritt.

Die Autoren der oben erwähnten Übersichtsarbeit versuchten, die Vorgänge bei einem Sturz etwas detaillierter zu beschreiben. Gestützt auf wissenschaftliche Studien zu Gleichgewichtsverlusten beim Gehen erklärten sie, dass man bei einem Stolpersturz die Arme reflexartig in die Sturzrichtung ausstreckt, um den Aufprall am Boden abfedern und dadurch den Kopf und Körper vor Verletzungen schützen zu können. Diese Abwehr ist nur wirksam, wenn zwischen dem ersten Bodenkontakt des Armes und dem Aufprall des Körpers möglichst viel Energie in Form von Muskelarbeit vernichtet wird. Weil der Arm bei einem Sturz nach vorne beim Bodenkontakt nach außen und bei einem Sturz auf die Seite an den Körper und nach innen gedrückt werden kann, können bei einem Sturz nach vorne die Subscapularissehne und bei einem Sturz zur Seite die Supraspinatus- und Infraspinatussehne und die zugehörigen Muskeln massiv exzentrisch belastet werden. Dass exzentrische Belastungen geeignet sind, einen Riss der Rotatorenmanschette zu verursachen, wurde auch in der versicherungsmedizinischen Literatur mehrfach festgehalten. Loew und Koautoren [5] notierten beispielsweise, dass eine passive forcierte Außen- oder Innenrotation bei anliegendem oder abgespreiztem Arm ein potenziell geeigneter Verletzungsmechanismus für die Entstehung einer Rotatorenmanschettenruptur sei. Sie notierten aber auch, dass ein einfacher Sturz nach vorne oder seitlich auf den Arm, ohne starke Verdrehung oder forcierte Adduktion, ein ungeeigneter Verletzungsmechanismus sei. Die auf den ersten Blick widersprüchlichen Aussagen sind wohl so zu interpretieren, dass man sich bei einem Sturz mit Abwehrversuch einen Sehnenriss zuziehen kann, nicht aber bei einem Sturz ohne Abwehrversuch.

Viele Patienten können sich nach einem Sturz nicht mehr genau daran erinnern, mit welchem Körperteil sie zuerst den Boden berührt hatten, und wie sie schließlich aufgeprallt waren. Manchmal berichten sie, dass sie die Arme ausgestreckt hatten und manchmal sagen sie, dass alles so schnell gegangen sei, dass sie ungebremst direkt auf die Schulter gestürzt seien. Letzteres traf auch bei dem hier beschriebenen Patienten zu. Er steht damit nicht allein da. Etwa ein Drittel aller Patientinnen und Patienten, bei denen wir nach einem Sturz eine Rotatorenmanschettenruptur diagnostizieren, geben an, direkt mit der Schulter aufgeprallt zu sein. Wir haben exemplarisch den oben beschriebenen Fall ausgewählt, weil die MRT-Aufnahmen und die intraoperativ festgestellte Einblutung in den Sehnenstummel keine Zweifel an der frischen Ruptur zulassen, und weil eine Schulterluxation aufgrund der fehlenden Impressionsfraktur am Humeruskopf als mögliche Ursache für die massive Ruptur ausgeschlossen werden kann. Es ist möglich, dass eine Biopsie gewisse degenerative Veränderungen im Sehnengewebe gezeigt hätte. Dies hätte jedoch nichts an der Unfallkausalität geändert. Alterungsprozesse sind normal und können in allen Geweben nachgewiesen werden. Sie führen jedoch nicht automatisch zu Symptomen oder einer Funktionsstörung der entsprechenden Organe. Wäre die massive Rotatorenmanschettenruptur im vorliegenden Fall schon vor dem Sturz vorhanden gewesen, hätte die MRT-Untersuchung eine fortgeschrittene Muskelatrophie gezeigt, und der Patient hätte keine schulterbelastenden Arbeiten über der Horizontalen ausführen können.

Der Pathomechanismus, welcher einer Sehnenläsion bei einem Sturz direkt auf die Schulter zugrunde liegt, ist noch nicht klar. Infrage kommt eine reflexartige massive isometrische Kontraktion der Rotatorenmanschettenmuskulatur bei anliegendem Arm. Dass es möglich ist, mit eigener Kraft eine Sehne vom Knochen abzureißen, wurde kürzlich von Lappen und Mitarbeitern [3] gezeigt. Sie analysierten im Internet publizierte Videoaufnahmen von akuten distalen Bizepssehnenrissen und fanden heraus, dass 93 % der 56 gefilmten Sehnenrisse beim Anspannen des Bizepsmuskels auftraten, in den meisten Fällen beim Gewichtheben („deadlift“) mit annähernd gestecktem Ellbogen und supiniertem Unterarm, in einer isometrischen und nicht in einer dynamischen Phase. Man kann sich vorstellen, dass dabei nicht alle Sehnenfasern gleichzeitig reißen, sondern dass der Riss an der schwächsten Stelle beginnt und sich dann lawinenartig auf die benachbarten Sehnenfasern ausdehnt.

Auch wenn der oben postulierte Pathomechanismus nicht bewiesen werden kann, zeigt das Beispiel doch deutlich, dass auch ein Sturz direkt auf die Schulter geeignet ist, eine Rotatorenmanschettenruptur zu verursachen. Es liegt auf der Hand, dass dabei nicht nur Massenrupturen, sondern auch kleinere Risse einzelner Sehnen oder Partialrupturen entstehen können. Die bisherige Unterteilung in geeignete und ungeeignete Stürze in der deutschsprachigen versicherungsmedizinischen Literatur ist willkürlich, weil es keine biomechanischen Studien dazu gibt, und weil die klinischen Studien zu einem anderen Schluss kommen. Die Unterteilung muss deshalb aufgegeben werden. Rotatorenmanschettenrupturen, welche nach einem Sturz diagnostiziert werden, sind in Analogie zur angelsächsischen Literatur unabhängig von der Sturzrichtung und der Armstellung als unfallbedingte Läsionen zu betrachten. Ausgenommen werden können Rupturen, bei denen der zugehörige Muskel auf zeitnah angefertigten MRT-Aufnahmen stark atrophiert ist und eine fettige Infiltration von mindestens Grad III nach Goutallier aufweist.
